# Enhanced supply of acetyl-CoA by exogenous pantothenate kinase promotes synthesis of poly(3-hydroxybutyrate)

**DOI:** 10.1186/s12934-023-02083-5

**Published:** 2023-04-20

**Authors:** Hirotaka Kudo, Sho Ono, Kenta Abe, Mami Matsuda, Tomohisa Hasunuma, Tomoyasu Nishizawa, Munehiko Asayama, Hirofumi Nishihara, Shigeru Chohnan

**Affiliations:** 1 Department of Food and Life Sciences, Ibaraki University College of Agriculture, 3-21-1 Chuo, Ami, Ibaraki 300-0393 Japan; 2 Graduate School of Science, Technology and Innovation, Kobe University, 1-1 Rokkodai, Nada, Kobe, 657-8501 Japan; 3 Engineering Biology Research Center, Kobe University, 1-1 Rokkodai, Nada, Kobe, 657-8501 Japan

**Keywords:** Coenzyme A, Acetyl-CoA, Pantothenate kinase, Poly(3-hydroxybutyrate), *Escherichia coli*, Pantothenic acid, β-Alanine

## Abstract

**Background:**

Coenzyme A (CoA) is a carrier of acyl groups. This cofactor is synthesized from pantothenic acid in five steps. The phosphorylation of pantothenate is catalyzed by pantothenate kinase (CoaA), which is a key step in the CoA biosynthetic pathway. To determine whether the enhancement of the CoA biosynthetic pathway is effective for producing useful substances, the effect of elevated acetyl-CoA levels resulting from the introduction of the exogenous *coaA* gene on poly(3-hydroxybutyrate) [P(3HB)] synthesis was determined in *Escherichia coli,* which express the genes necessary for cyanobacterial polyhydroxyalkanoate synthesis (*phaABEC*).

**Results:**

*E. coli* containing the *coaA* gene in addition to the *pha* genes accumulated more P(3HB) compared with the transformant containing the *pha* genes alone. P(3HB) production was enhanced by precursor addition, with P(3HB) content increasing from 18.4% (w/w) to 29.0% in the presence of 0.5 mM pantothenate and 16.3%–28.2% by adding 0.5 mM β-alanine. Strains expressing the exogenous *coaA* in the presence of precursors contained acetyl-CoA in excess of 1 nmol/mg of dry cell wt, which promoted the reaction toward P(3HB) formation. The amount of acetate exported into the medium was three times lower in the cells carrying exogenous *coaA* and *pha* genes than in the cells carrying *pha* genes alone. This was attributed to significantly enlarging the intracellular pool size of CoA, which is the recipient of acetic acid and is advantageous for microbial production of value-added materials.

**Conclusions:**

Enhancing the CoA biosynthetic pathway with exogenous CoaA was effective at increasing P(3HB) production. Supplementing the medium with pantothenate facilitated the accumulation of P(3HB). β-Alanine was able to replace the efficacy of adding pantothenate.

**Supplementary Information:**

The online version contains supplementary material available at 10.1186/s12934-023-02083-5.

## Background

Coenzyme A (CoA) is an important cofactor in many biological reactions in addition to ATP and NAD(P)H. This molecule functions as a carrier for acyl groups and is synthesized from pantothenic acid in five enzymatic steps [1–3]. Pantothenate kinase (CoaA) catalyzes the phosphorylation of pantothenic acid in the first step. It is an important enzyme that characterizes the CoA biosynthetic pathway. Bacterial CoaAs are classified into three groups based on their primary structure [3]. Prokaryotic type I CoaA found in *Escherichia coli* pantothenate kinase is inhibited by free CoA and acyl-CoAs [4]. Prokaryotic type II CoaA, found exclusively in staphylococci, is refractory to feedback inhibition by CoA and its derivatives [5, 6]. Prokaryotic type III CoaAs from *Helicobacter pylori* and *Pseudomonas aeruginosa* are also not susceptible to end-product inhibition, but they require monovalent cations, such as K^+^ and NH_4_^+^, for their enzymatic activities [7, 8]. The bacterial CoA pool consists mainly of CoA, acetyl-CoA, and malonyl-CoA, of which acetyl-CoA is the major component [9, 10]. Facultative anaerobes contain larger CoA pools compared with aerobic bacteria [11]. To produce value-added compounds, we increased intracellular acetyl-CoA and malonyl-CoA levels using *E. coli* as a model microorganism. Expression of the gene encoding type III CoaA from *P. putida* resulted in a 2.8-fold increase in the size of the CoA pool, with acetyl-CoA accounting for 88% of the total [12]. Increased malonyl-CoA levels were achieved by co-expressing the acetyl-CoA carboxylase (Acc) genes from *Corynebacterium glutamicum* along with the *coaA* gene, which resulted in a greater than sevenfold increase in fatty acid content [13, 14].

**Fig. 1:**
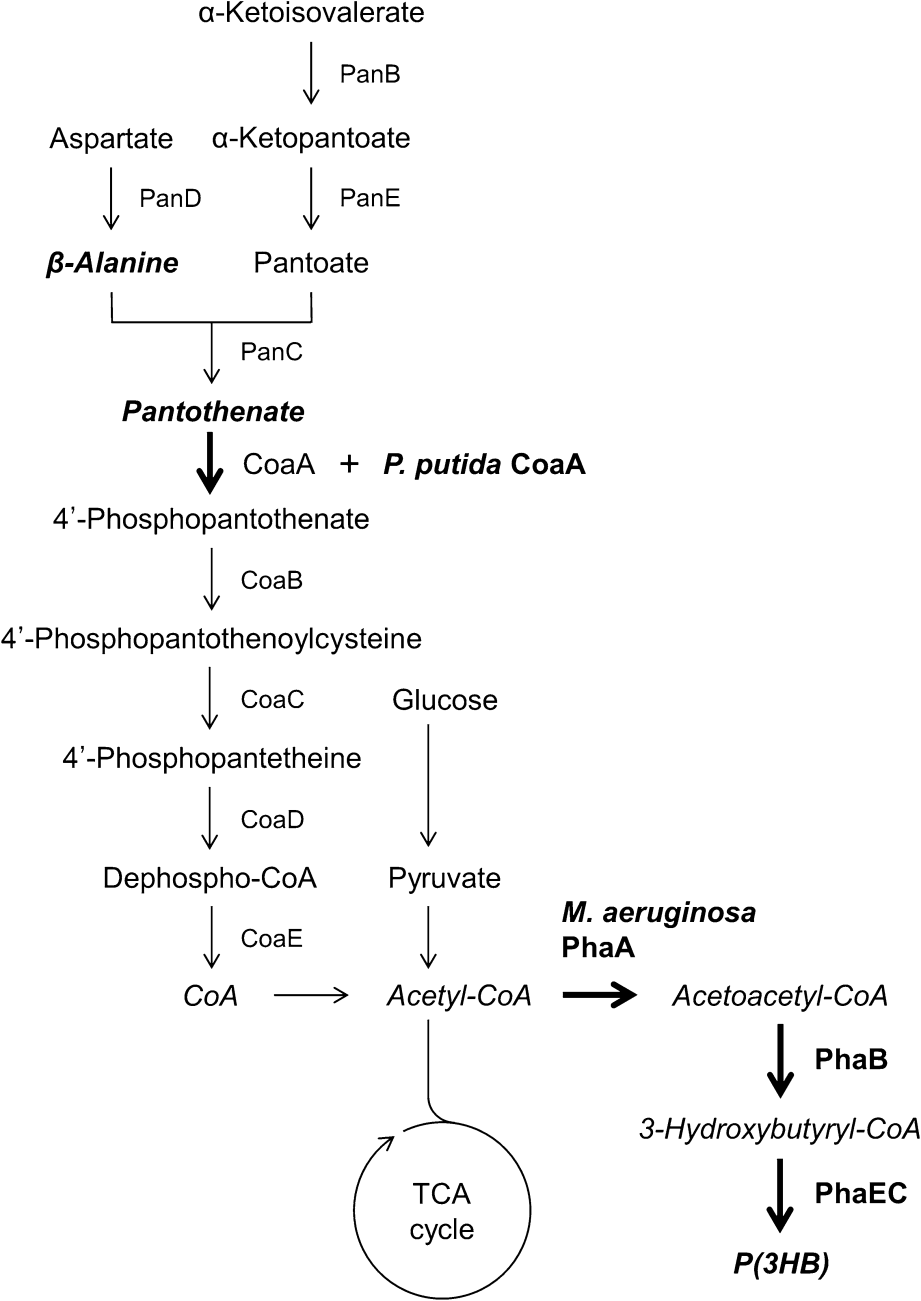
Metabolic pathway for poly(3-hydroxybutyrate) production through acetyl-CoA in *E. coli* cells. PanB, ketopantoate hydroxymethyltransferase; PanC, pantothenate synthetase; PanD, L-aspartate-α-decarboxylase; PanE, ketopantoate reductase; CoaA, pantothenate kinase; CoaB, 4'-phosphopantothenoylcysteine synthetase; CoaC, 4'-phosphopantothenoylcysteine decarboxylase; CoaD, phosphopantetheine adenylyltransferase; CoaE, dephospho-CoA kinase from *E. coli*. Exogenous genes encoding the enzymes indicated in bold letters were introduced into *E. coli* cells. *P. putida* CoaA, the bacterial type III pantothenate kinase from *P. putida*; *M. aeruginosa* PhaA, β-ketothiolase; PhaB, acetoacetyl-CoA reductase; PhaEC, polyhydroxyalkanoate synthase from *M. aeruginosa*

Acetyl-CoA provides an acetyl group in numerous metabolic pathways. Poly(3-hydroxybutyrate) [P(3HB)], a biodegradable plastic, is synthesized from acetyl-CoA. Canonical P(3HB) biosynthesis starts with the Claisen condensation of two acetyl-CoA molecules by β-ketothiolase. The produced acetoacetyl-CoA is then reduced to 3-hydroxybutylyl-CoA by acetoacetyl-CoA reductase using NADPH. Finally, P(3HB) is synthesized by a polymerization reaction catalyzed by polyhydroxyalkanoate (PHA) synthase [15, 16]. Although P(3HB) is produced by several microorganisms, P(3HB) biosynthesis by *Cupriavidus necator* H16 (formerly *Ralstonia eutropha*) has been extensively studied [17]. As photosynthetic bacteria can also synthesize P(3HB) via the Calvin cycle by fixing carbon dioxide, which is one of the compounds responsible for global warming, cyanobacteria could be among the key producers of this bioplastic [18, 19]. *Synechocystis* sp. PCC 6803 is a widely studied species because it grows fast and can be genetically modified easily. It was shown to have a P(3HB) content of 4.1% (w/w) under light irradiation [20]. Previously, we engineered *Synechocystis* sp. to carry a plasmid containing the genes for PHA synthesis, *phaABEC* from *Microcystis aeruginosa* (Fig. 1). The cells accumulated approximately 7% (w/w) P(3HB) with a 12-fold higher productivity under nitrogen depletion and light irradiation [21]. However, metabolome analysis using capillary electrophoresis-mass spectrometry (CE-MS) indicated that the cellular acetoacetyl-CoA and 3-hydroxybutyryl-CoA were undetectable or extremely low. The amount of acetyl-CoA, a substrate for P(3HB) biosynthesis, decreased to one-fifth compared with that of the cells grown in the presence of sodium nitrate. It was concluded that enlargement of the acetyl-CoA pool size may lead to an overproduction of P(3HB) in cyanobacteria.

Cofactor engineering using NADPH is effective in P(3HB) and 3HB production in *E. coli*, suggesting an improvement in the reduction of acetoacetyl-CoA to 3-hydroxybytyryl-CoA by acetoacetyl-CoA reductase, which catalyzes the second reaction in the P(3HB) biosynthetic pathway [22–24]. In the present study, we determined the effectiveness of increasing intracellular acetyl-CoA by exogenous *coaA* expression (i.e*.*, CoA-cofactor engineering), on P(3HB) production using *E. coli* expressing the cyanobacterial *phaABEC* genes (Fig. 1). Although a small increase in P(3HB) was observed by the introduction of *coaA* gene alone, the addition of CoA precursors, which are essential for increasing the intracellular CoA pool size, to the medium further enhanced P(3HB) production, with an approximate twofold P(3HB) content, 30% (w/w) of dry cell weight. Furthermore, a suppressive effect on acetate formation from acetyl-CoA was also observed through an increase in intracellular CoA levels. This indicates that CoA-cofactor engineering is highly effective for substance production.

## Results and discussion

### P(3HB) synthesis by PHA biosynthesis genes from *M. aeruginosa*

**Fig. 2:**
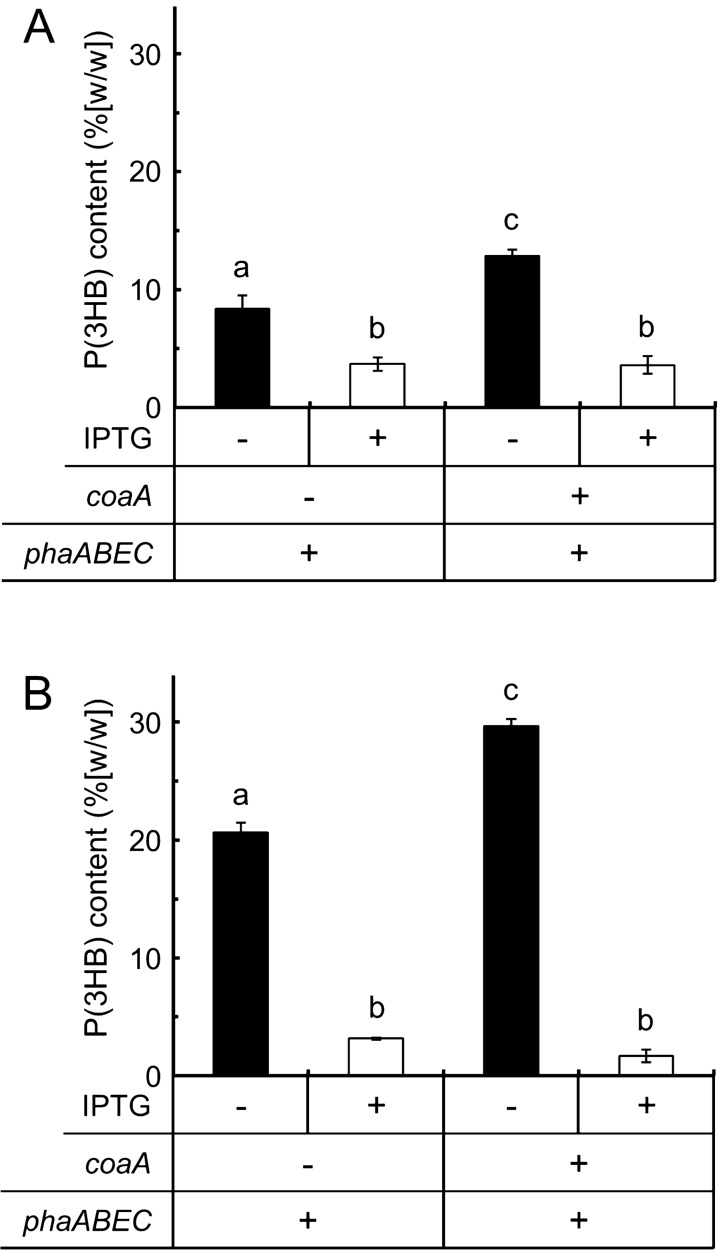
P(3HB) contents in *E. coli* cells carrying the *pha* genes from *M. aeruginosa*. The transformants of *E. coli* JM109/pSTV28 + pQE-Ma-phaABEC and JM109/pSTV-Pp-coaA + pQE-Ma-phaABEC were cultivated aerobically in 30 mL of M9 minimal medium supplemented with 5 mM pantothenate and 2% (w/v) glucose in the absence (solid bar) or presence (open bar) of 0.1 mM IPTG for 48 h at 30 °C (**A**) and 37 °C (**B**). P(3HB) content was determined as 3HB methyl esters using GC-FID. The significance of the differences was analyzed by Tukey's HSD test (*p* < 0.01). The data are shown as the mean ± SD (*n* = 3)

The four transformants for P(3HB) production were prepared using *E. coli* JM109 as a host: *E. coli* JM109/pSTV28 + pQE-60, JM109/pSTV-Pp-coaA + pQE-60, JM109/pSTV28 + pQE-Ma-phaABEC, and JM109/pSTV-Pp-coaA + pQE-Ma-phaABEC. After verifying the plasmids carried by each transformant (Additional file 1: Figure S1), the effect of IPTG induction on P(3HB) production by the recombinants was examined using minimal medium containing 2% (w/v) glucose and 5 mM pantothenate, which is an indispensable precursor of CoA to expand the size of intracellular CoA pools in *E. coli* through exogenous *coaA* expression [12]. The transformants carrying the empty plasmid without *phaABEC* (i.e., JM109/pSTV28 + pQE-60 and JM109/pSTV-Pp-coaA + pQE-60) failed to accumulate P(3HB). In contrast, P(3HB) production was clearly observed in the two transformants with pQE-Ma-phaABEC (Fig. 2). Both transformants of JM109/pSTV28 + pQE-Ma-phaABEC and JM109/pSTV-Pp-coaA + pQE-Ma-phaABEC accumulated larger amounts of P(3HB) in the absence of IPTG than those who were treated with IPTG. The lower production of cellular P(3HB) following IPTG induction appeared to result from poor growth during the early stage of culture (Additional file 1: Figure S2) and the loss of pQE-Ma-phaABEC from the transformants (Additional file 1: Table S1). When grown at 37 °C in the presence of 0.1 mM IPTG, pQE-Ma-phaABEC was lost in 28% of the JM109/pSTV28 + pQE-Ma-phaABEC transformants and in 59% of the JM109/pSTV-Pp-coaA + pQE-Ma-phaABEC transformants. In contrast, the *coaA* expression plasmid, pSTV-Pp-coaA, was stable even at 37 °C cultivation with IPTG. Overall, the introduction of the exogenous *coaA* gene encoding the type III pantothenate kinase from *P. putida*, which is capable of enlarging the size of the CoA pool [12], increased the accumulation of P(3HB) from 8.36 to 12.8% (w/w) in 30 °C cultivation (Fig. 2A) and from 20.7 to 29.7% (w/w) at 37 °C (Fig. 2B) without IPTG induction, respectively, showing that the leaky expression of *coaA* and/or *phaABEC* genes is sufficient and preferable to verify the effectiveness of introducing the *coaA* gene.

### Effect of addition of CoA precursors on P(3HB) synthesis

**Fig. 3:**
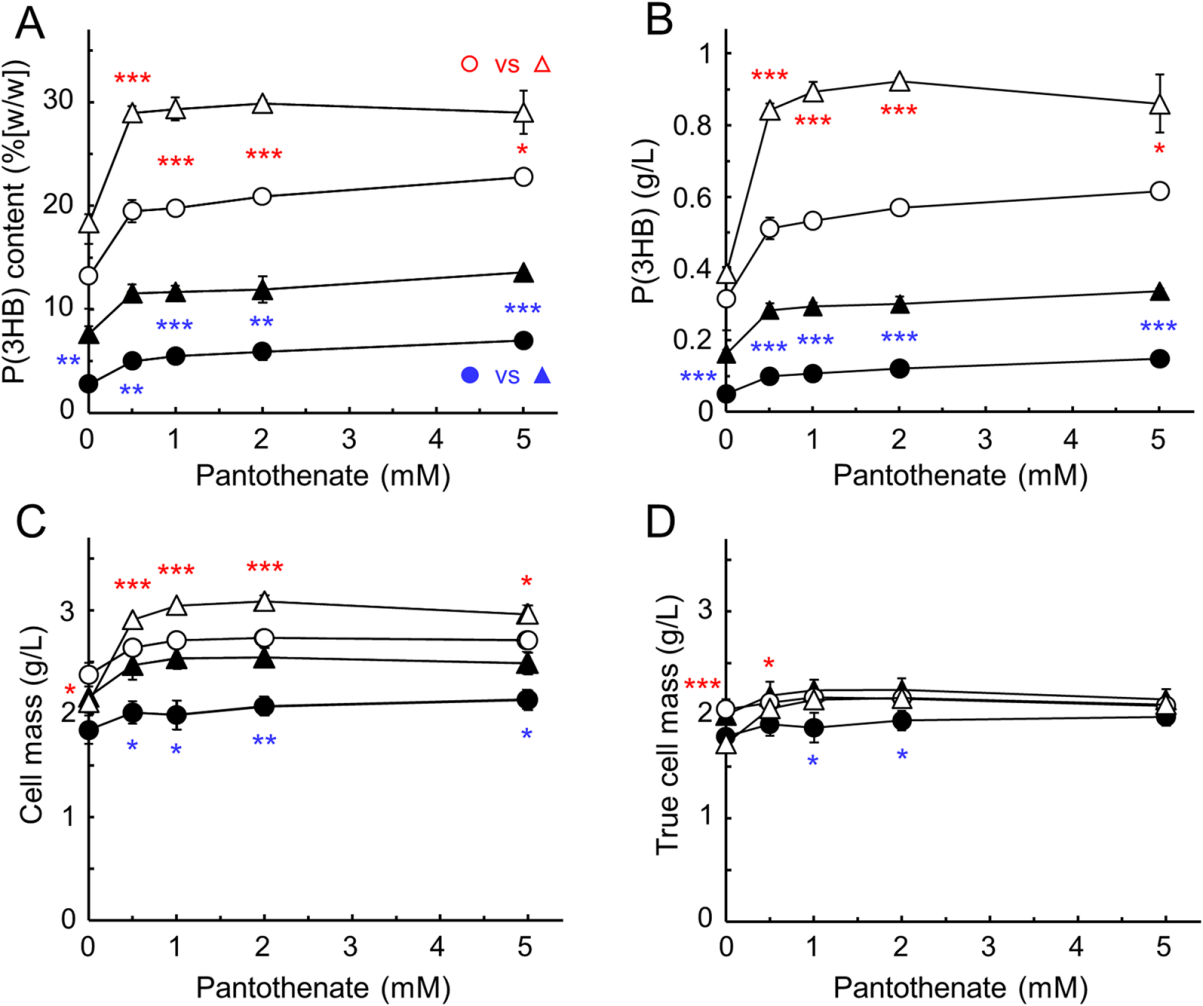
Effect of pantothenate on P(3HB) production in *E. coli* containing the exogenous *coaA* gene. The transformants of *E. coli* JM109/pSTV28 + pQE-Ma-phaABEC (circles) and JM109/pSTV-Pp-coaA + pQE-Ma-phaABEC (triangles) were grown in 30 mL of minimal medium containing 2% (w/v) glucose and various concentrations of pantothenate without 0.1 mM IPTG for 48 h at 30 °C (closed symbols) or 37 °C (open symbols). Cellular P(3HB) was converted into 3HB methyl esters and measured using GC-FID. The P(3HB) content is indicated as % (w/w) of dry cell weight (**A**) and g/L (**B**). Cell mass is expressed in g of dry cell weight per 1 litter (**C**). True cell mass was calculated by subtracting P(3HB) from cell mass (**D**). The significant difference between *coaA* – and + at each concentration of pantothenate was compared using an unpaired Student's *t*-test: *, *p* < 0.05; **, *p* < 0.01; ***, *p* < 0.001. The data are shown as the mean ± SD (*n* = 3)

The introduction of exogenous *coaA* gene was capable of enlarging the size of intracellular CoA pools in *E. coli* and acetyl-CoA accounted for over 70% of the CoA pool content at least [12]. The addition of pantothenate to the medium was essential, whereas β-alanine could also be substituted. To determine the relationship between acetyl-CoA supply and P(3HB) production, the effect of adding these precursors to P(3HB) production was analyzed. The effect of pantothenate addition was evaluated at 30 °C and at 37 °C cultivation temperatures (Fig. 3A and B). In terms of P(3HB) production in the absence of pantothenate, the introduction of the *coaA* gene alone slightly increased the P(3HB) level from 13.2% to 18.4% (w/w) of dry cell wt at 37 °C culture and from 2.77% to 7.61% (w/w) of dry cell wt at 30 °C culture. These results imply that *E. coli* produced more pantothenic acid than required for its CoA biosynthesis [25], and the excess pantothenate was readily converted to acetyl-CoA via enhanced CoA biosynthesis by the foreign CoaA. When incubated at 37 °C, larger amounts of P(3HB) were produced compared with that at 30 °C. Even the transformants with the *pha* genes alone grown at 37 °C produced more P(3HB) compared with the strain carrying *P. putida coaA* together with the *pha* genes grown at 30 °C at each pantothenate concentration. The intracellular P(3HB) content of transformants containing both the *coaA* and *phaABEC* genes increased from 18.4% (w/w) of dry cell wt to 29.0% with the addition of 0.5 mM pantothenate. At concentrations above 0.5 mM, the accumulation was almost similar at 29% (w/w), which corresponds to a production of approximately 0.9 g/L culture. *E. coli* expresses pantothenate permease (PanF) and actively incorporates extracellular pantothenate [26–28]. Surprisingly, the strain without the exogenous *coaA* gene, JM109/pSTV28 + pQE-Ma-phaABEC, also responded to pantothenate, with a yield increasing from 13.2% (w/w) to 19.5% (w/w). This strain exhibited a maximum accumulation of 22.8% (w/w) P(3HB) following the addition of 5 mM pantothenate. This result reflects the fact that adding pantothenate to the medium doubled the size of the intracellular CoA pool even in *E. coli* transformants without exogenous *coaA* genes [12]. In addition, cell masses, meaning sum of true cell mass and P(3HB) content, were increased by the addition of pantothenate and showed the highest value in the JM109/pSTV-Pp-coaA + pQE-Ma-phaABEC strain (Fig. 3C). Because these values reflect the P(3HB) content, the true cell masses of all transformants were approximately 2.1 g of dry cell wt/L, whereas that of JM109/pSTV 28 + pQE-Ma-phaABEC at 30 °C cultivation was slightly lower at approximately 1.9 g/L (Fig. 3D). Overall, in *E. coli* cells containing the genes for PHA synthesis from *M. aeriginosa*, the introduction of the foreign type III *coaA* gene from *P. putida* alone increases P(3HB) production. Furthermore, the addition of a CoA precursor required to increase the size of the CoA pools by CoaA induced the function of *pha* gene products, which indicates that the enhanced supply of acetyl-CoA is effective for P(3HB) synthesis. These results were obtained by incubating at 37 °C and 30 °C.

**Fig. 4:**
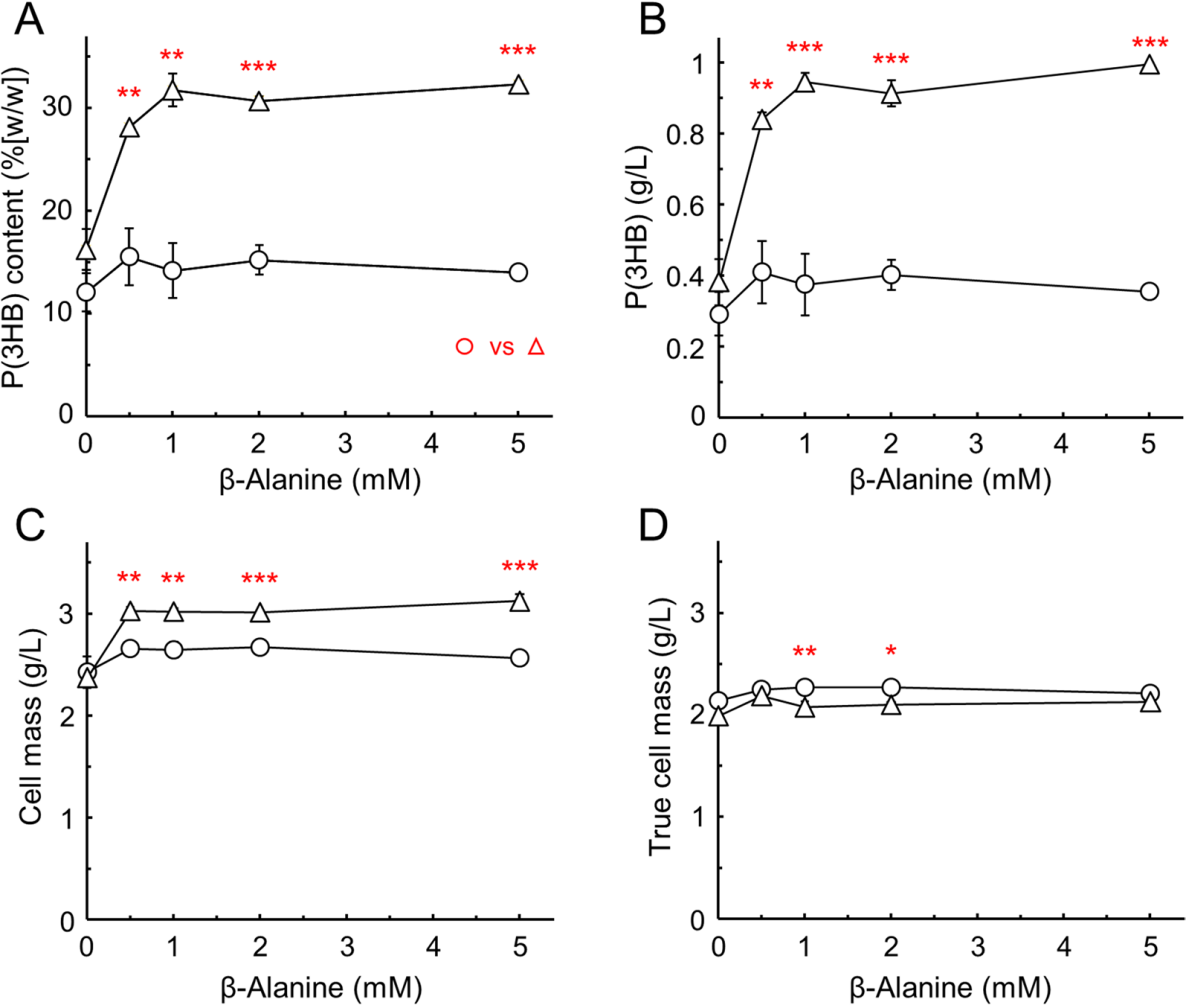
Effect of β-alanine addition on P(3HB) production in *E. coli* transformants. The transformants of *E. coli* JM109/pSTV28 + pQE-Ma-phaABEC (open circles) and JM109/pSTV-Pp-coaA + pQE-Ma-phaABEC (open triangles) were cultivated in 30 mL of minimal medium containing various concentrations of β-alanine for 48 h at 37 °C. The methyl ester prepared from P(3HB) was analyzed using GC-FID. The panels **A**, **B**, **C**, and **D** are the same as Fig. 3. The significant difference between *coaA* – and + at each concentration of β-alanine was compared with an unpaired Student's *t*-test: *, *p* < 0.05; **, *p* < 0.01; ***, *p* < 0.001. The data are shown as the mean ± SD (*n* = 3)

With respect to the supply of CoA precursors, β-alanine could substitute for pantothenate and increased the CoA pool size to 70% of the level generated in the presence of 5 mM pantothenate [12]. Therefore, the effect of β-alanine on P(3HB) production was evaluated (Fig. 4A and B). As expected, the addition of β-alanine increased the production of P(3HB) in the strains carrying both genes, reaching approximately 30% (w/w), which is similar to the value obtained from pantothenate addition. Even in the strain expressing *pha* genes only, the addition of β-alanine increased P(3HB) production to approximately 2%, although not high as that of pantothenate. The amino acid carrier CycA is responsible for the incorporation of external β-alanine into *E. coli* cells [29]. Even in the *E. coli* strain overexpressing its own *panD,* which codes for L-aspartate-α-decarboxylase and catalyzes the decarboxylation of L-aspartic acid to produce β-alanine, the addition of β-alanine was effective for pantothenic acid production [30]. Additionally, our previous study demonstrated that L-aspartic acid or pantoic acid in the medium did not affect the size or composition of CoA pools in *E. coli* harboring exogenous *coaA* [12]. Pantoate, which is another substrate for pantothenate synthase (PanC) to provide pantothenate, is produced in 15-fold greater amounts compared with that required to maintain intracellular CoA and acyl-CoA concentrations [25]. Taken together, the supply of β-alanine is a rate-limiting step, particularly for expanding the intracellular CoA pool sizes with the additional CoaA. This issue can be resolved by enhancing PanD activity; that is, through expression of PanD from *Corynebacterium glutamicum*. Unlike *E. coli* PanD, the decarboxylase from *C. glutamicum* is capable of filling the intracellular pantothenate pool of *E. coli* without β-alanine supplementation [31].

### Time-course analysis of P(3HB) production

**Fig. 5:**
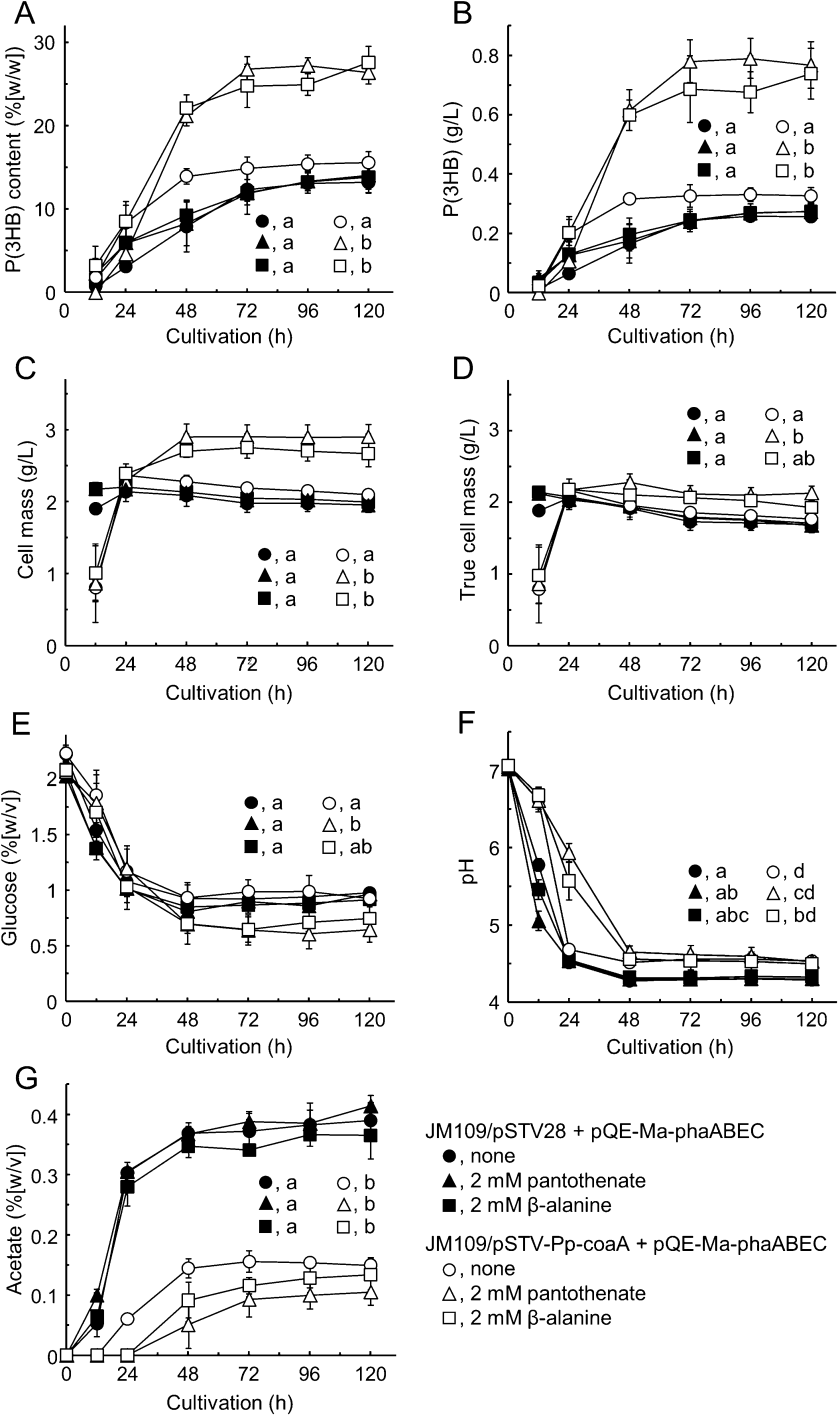
Accumulation of P(3HB) by enhanced acetyl-CoA supply in *E. coli*. The transformants of *E. coli* JM109/pSTV28 + pQE-Ma-phaABEC (closed symbols) and JM109/pSTV-Pp-coaA + pQE-Ma-phaABEC (open symbols) were aerobically cultivated in 150 mL of M9 minimal medium in the absence (circles) or presence of 2 mM pantothenate (triangles) or 2 mM β-alanine (squares) for 120 h at 37 °C. Panels **A**–**D** show P(3HB) content and cell mass as described in Fig. 3. Panels **E**, **F**, and **G** indicate residual glucose, pH, and acetate in the culture media, respectively. The significant differences among the six experimental groups at 120 h of cultivation were analyzed by Turkey’s HSD test (*p* < 0.05). All data are shown as the mean ± SD (*n* = 3)

The relationship between P(3HB) production and cultivation time was determined at 37 °C for 120 h using a 500 mL baffled Erlenmeyer flask containing 150 mL of minimal medium (Fig. 5). Cellular P(3HB) accumulation in the transformants with *coaA* and *phaABEC* was significantly increased in the presence of either precursor at 2 mM, the concentration that could fully contribute to P(3HB) production. After a 72-h cultivation, the content reached nearly maximal levels of 26.3% (w/w) in the presence of pantothenate and 24.8% (w/w) in the presence of β-alanine, which corresponds to 0.780 g/L and 0.686 g/L, respectively (Fig. 5A and B). The accumulation of P(3HB) in the JM109/pSTV-Pp-coaA + pQE-Ma-phaABEC strain grown in medium containing neither precursor was approximately 15% (w/w) after 120 h of cultivation. This result was not significantly different from that observed in the transformants with *phaABEC* alone, whereas the intracellular product reached a maximum level after 48 h, clearly producing it faster compared with the strain containing only the *pha* genes. Thus, the addition of the CoA precursor to the culture medium of the transformants carrying the *coaA* gene, along with the *pha* genes, enhanced P(3HB) production in the scaled-up experiment. Moreover, the *pha* gene-only strain produced slightly more product through the addition of precursors during a 48-h cultivation as shown in Figs. 3 and 4.

As shown in Fig. 5C, the growth of exogenous *coaA*-bearing bacteria in a 12-h cultivation was delayed. The cell masses were increased after 48 h when more P(3HB) had accumulated from precursor addition, whereas there was no significant difference in their true cell masses (Fig. 5D).

The residual glucose in the *coaA*-carrying strain grown without the precursors, approximately 0.9% (w/v), was comparable to that in the strains lacking the *coaA* gene. However, when strains were grown in the presence of pantothenate or β-alanine, the residual glucose levels were approximately 0.7% (w/v), indicating increased glucose consumption (Fig. 5E). Furthermore, the pH values of the medium after 48 h were slightly high in the transformants with the additional *coaA* gene (Fig. 5F). Interestingly, acetate efflux from *E. coli* cells carrying the prokaryotic type III *coaA* gene into the medium was reduced to one-third of that in the cells without the extra *coaA* gene (Fig. 5G). Hence, the decrease in pH was thought to be due to the accumulation of acetate. Acetate production was minimal in the strain cultured in the presence of 2 mM pantothenate at approximately 0.1% (w/v), which corresponds to 17 mM. These results indicate that the intracellular CoA increases by *coaA* gene overexpression, which prevents acetate wastage and helps in effective P(3HB) production. There are two pathways for the synthesis of acetate in *E. coli* aerobically: from pyruvate oxidase (PoxB) and from acetyl-CoA by acetate kinase (AckA) and phosphate acetyltransferase (Pta) [32–34]. The deletion of *ackA*-*pta* alone dramatically decreased acetate production under anaerobic conditions [35]. In contrast, disruption of either *ackA*-*pta* or poxB alone had no effect on acetate formation in *E. coli* under aerobic conditions, whereas the disruption of both was necessary to avoid substrate waste [35, 36]. The expression of the exogenous type III *coaA* gene appears to contribute to the storage of acetyl-CoA from glycolysis as well as increasing the supply of acetyl group.

### Relationship between P(3HB) synthesis and its intermediates

**Fig. 6:**
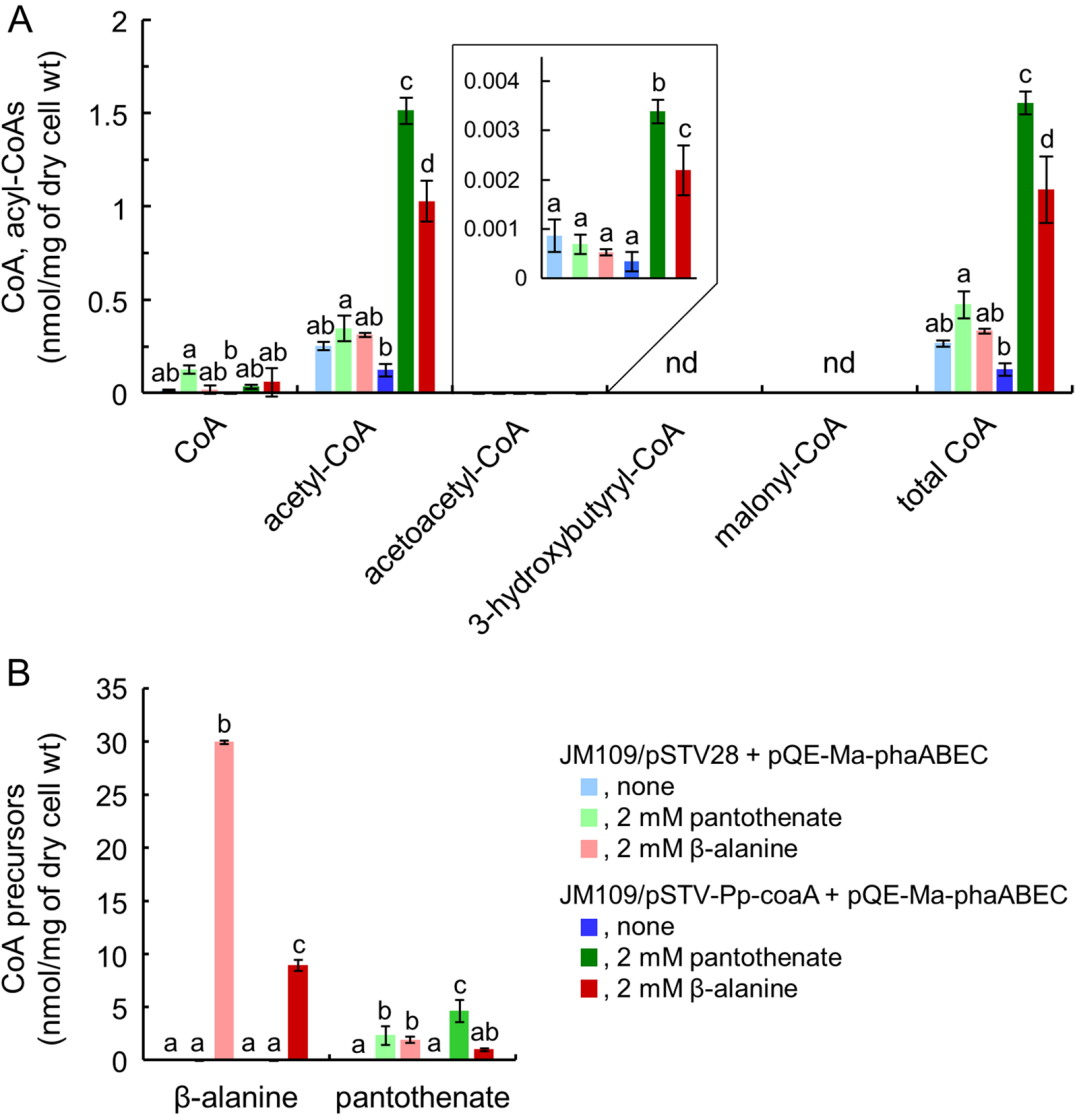
Analysis of metabolic intermediates during P(3HB) accumulation. The transformants of *E. coli* JM109/pSTV28 + pQE-Ma-phaABEC (pale colors) and JM109/pSTV-Pp-coaA + pQE-Ma-phaABEC (dark colors) were aerobically cultivated in 30 mL of M9 minimal medium in the absence (blue) or presence of 2 mM pantothenate (green) or 2 mM β-alanine (red) for 32 h at 37 °C. Intracellular CoA and its derivatives (**A**) and CoA precursors (**B**) were measured by CE-MS. The significant differences were analyzed by Turkey’s HSD test (*p* < 0.05). All data are shown as the mean ± SD (*n* = 3). nd, not detected

To confirm the increase in intracellular CoA due to overexpression of the *coaA* gene and feeding of CoA precursors, metabolic intermediates of the CoA biosynthetic pathway at 32 h of cultivation were analyzed via CE-MS. P(3HB) synthesis in transformants cultured for 32 h was expected to be fully active or in the initial stages of decline; however, P(3HB) production would progress in both cases. Intracellular acetyl-CoA in *E. coli* cells carrying the exogenous *coaA* gene from *P. putida* was increased by adding CoA precursors (Fig. 6A). In the presence of 2 mM pantothenate, the acetyl-CoA content in the JM109/pSTV-Pp-coaA + pQE-Ma-phaABEC strain exhibited a 12-fold increase to reach a maximum value of 1.51 nmol/mg of dry cell wt (Fig. 6A). Even with the addition of β-alanine, the size of the acetyl-CoA pool expanded to 8.2-fold, which corresponds to 68% of that with pantothenate. The effect of β-alanine addition on the intracellular CoA pool size in the transformants producing P(3HB) was consistent with that obtained with *E. coli* W3110 [12]. As mentioned above, increased P(3HB) production was observed in strains harboring the exogenous *coaA* gene following the addition of the precursors, which suggests that the increased acetyl-CoA content enhanced P(3HB) synthesis. The acetoacetyl-CoA content synthesized from acetyl-CoA by β-ketothiolase (PhaA) was much lower compared with that of acetyl-CoA, less than 0.004 nmol/mg of dry cell wt, but reflected the cellular concentration of acetyl-CoA. This intermediate concentration was highest in the JM109/pSTV-Pp-coaA + pQE-Ma-phaABEC strain grown with pantothenate, followed by cells grown in the presence of β-alanine. 3-Hydroxybutyryl-CoA, which is the substrate of the polymerization reaction by PHA synthase (PhaEC) belonging to class III [37], was not detected in any of the transformants. These results imply that the initial step catalyzed by PhaA is the rate-limiting step in P(3HB) synthesis of *M. aeruginosa*, although the second reaction by acetoacetyl-CoA reductase (PhaB) and the third reaction by PhaEC proceeded rapidly to produce a polymer of 3-hydroxybutyrate. *Synechocystis* sp. PCC 6803 harboring the *pha* genes from *M. aeruginosa* also yielded a similar composition of CoA derivatives [21]. In addition, this phenomenon was observed in an in vitro reconstitution study of *Cupriavidus necator* H16 *pha* gene products expressed in *E. coli* [38]. Taken together, PhaA is a key determinant of the bioplastic production with these bacteria. Essentially, the condensation reaction of acetyl-CoA is thermodynamically unfavorable and is not oriented toward the formation of acetoacetyl-CoA [39]. Therefore, it makes sense to use CoaA to fill the intracellular acetyl-CoA pool and apply pressure in the direction of acetoacetyl-CoA production.

Pantothenate and β-alanine are incorporated into cells by PanF and CycA, respectively [26, 27, 29]. As shown in Fig. 6B, these CoA precursors added to the medium were efficiently taken up into the cells. In particular, the intracellular concentrations of β-alanine were much higher and the precursor was taken up into cells at 30.0 nmol/mg of dry cell wt in the JM109/pSTV 28 + pQE-Ma-phaABEC strain and at 8.95 nmol/mg in the JM109/pSTV-Pp-coaA + pQE-Ma-phaABEC strain. These incorporated substances were certainly converted to pantothenic acid by PanC, which was present in the cells at greater than 1 nmol/mg of dry cell wt.

## Conclusions

The introduction of the gene encoding the prokaryotic type III pantothenate kinase, which is insensitive to feedback inhibition by CoA and acyl-CoAs, clearly accelerated P(3HB) production in *E. coli* harboring the cyanobacterial *pha* genes. This effect was enhanced by the addition of the CoA precursor, pantothenate or β-alanine, into the medium, which suggests that the elevated acetyl-CoA levels boosted P(3HB) synthesis. In fact, the introduction of the *coaA* gene and the addition of CoA precursors greatly expanded the size of the CoA pool, approximately 95% of which was occupied by acetyl-CoA. Uptake of β-alanine into cells through the CycA transporter was much more efficient compared with pantothenic acid uptake by PanF, whereas it did not elevate intracellular acetyl-CoA levels as much as pantothenic acid addition. However, in P(3HB) production using the *pha* genes from *M. aeruginosa*, the effect of exogenous *coaA* gene introduction may be sufficiently induced even with the addition of β-alanine. Another advantage of the CoA pools enlarged by the additional CoaA was the suppression of acetate formation from acetyl-CoA, which is the substrate for P(3HB) biosynthesis. Thus, we demonstrated the effectiveness of CoA-cofactor engineering for P(3HB) production in the model organism *E. coli*.

Although using a different approach, it was observed that acetic acid and isopropanol were produced 7- and ninefold higher, respectively, in *Synechococcus elongatus* PCC 7942 overexpressing their own pyruvate dehydrogenase complex genes, the gene products of which catalyze the conversion of pyruvate to acetyl-CoA [40]. Therefore, in the near future, we will aim to achieve relatively high P(3HB) production by enhancing the CoA and/or pantothenic acid biosynthetic pathways in cyanobacteria.

## Materials and methods

### Materials

The *Escherichia coli* JM109 competent cells, PrimeSTAR HS DNA polymerase, EmeraldAmp MAX PCR Master Mix, In-Fusion HD cloning kit, and *Nco*I were purchased from Takara Bio, Inc. (Shiga, Japan). The pQE-60 plasmid was obtained from Qiagen GmbH (Hilden, Germany). Poly(*R*)-3-hydroxybutyric acid, ( ±)3-hydroxybutyric acid (3HB) sodium salt and L-methionine sulfone were purchased from Sigma-Aldrich, Inc. (St. Louis, MO, USA). Acetone, sulfuric acid, methanol, sodium D-pantothenate, β-alanine, ampicillin sodium salt (Ap), chloramphenicol (Cm), isopropyl β-D-thiogalactopyranoside (IPTG), ethanol for HPLC, and chloroform for HPLC were purchased from FUJIFILM Wako Pure Chemical Corporation (Osaka, Japan). Oligonucleotides for PCR and DNA sequencing were purchased from Eurofins Genomics K.K. (Tokyo, Japan). PIPES sesquisogium and D-camphor-10-sulfonic acid were purchased from Dojindo Laboratories Co., Ltd. (Kumamoto, Japan) and Nacalai Tesque, Inc. (Kyoto, Japan), respectively. Amicon Ultra-0.5 Centrifugal Filter Units (3 kDa) were obtained from Merck KGaA (Darmstadt, Germany). All other chemicals were of reagent grade.

### Bacterial strains, plasmids, and culture conditions

The bacterial strains and plasmids used in this study are listed in Table 1. The expression plasmid for CoaA from *P. putida*, pSTV-Pp-coaA, was constructed in a previous study [12]. The gene cluster containing of *phaA*, *phaB*, *phaE*, and *phaC* from *Microcystis aeruginosa* NIES-843 (GenBank accession no. AP009552) was amplified by performing PCR using the Ma-phaA-gF and Ma-phaC-gR primer set (Additional file 1: Table S2). The resulting DNA fragment was then cloned downstream of the T5 promotor of pQE-60, which had previously been digested using *Nco*I, to generate pQE-Ma-phaABEC (Additional file 1: Figure S1). The nucleotide sequence of the cloned gene was determined using the BigDye terminator v3.1 Cycle Sequencing Kit with primers (Additional file 1: Table S2) on an ABI PRISM 3130xl Genetic Analyzer (Applied Biosystems Japan Ltd., Tokyo, Japan), as shown in Figure S3. The plasmid and pSTV-Pp-coaA were transformed into *E. coli* JM109 and the resulting recombinants were analyzed for P(3HB) production.

**Table 1:** Strains and plasmids used in this work

Strain or plasmid	Relevant genotype or characteristic	Source or reference
Strain		
*Pseudomonas putida* JCM 20,089	Template DNA for PCR	JCM^a^
*Microcystis aeruginosa* NIES-843	Template DNA for PCR	NIES^b^
*Escherichia coli* JM109	*recA1*, *endA1*, *gyrA96*, *thi*-*1*, *hsdR17*(*r*_*k*_^*−*^*m*_*k*_^+^), *e14*^−^(*mcrA*^−^), *supE44*, *relA1*, Δ(*lac-proAB*)*/*F'[*traD36*, *proAB*^+^, *lacI*^q^, *lacZ*Δ*M15*]	Takara
Plasmid		
pQE-60	Ap^r^, ColE1, expression vector, T5 promoter	Qiagen
pQE-Ma-phaABEC	Ap^r^, pQE-60 carrying *pha* gene cluster of *phaA, phaB, phaE and phaC* from *M. aeruginosa* under T5 promoter	This work
pSTV28	Cm^r^, p15A, cloning vector, *lac* promoter	Takara
pSTV-Pp-coaA	Cm^r^, pSTV28 carrying *coaA* from *P. putida* under *lac* promoter	12

^a^Japan Collection of Microorganisms

^b^The National Institute for Environmental Studies

The transformants were cultivated aerobically in Luria–Bertani broth supplemented with 100 μg/mL Ap and 25 μg/mL Cm at 30 °C overnight. Preculture solution (300 μL) was added to 30 mL of M9 minimal medium supplemented with the trace elements listed below, 2% (w/v) glucose, 5 mM pantothenate, and appropriate antibiotics in 200 mL baffled Erlenmeyer flasks. The transformants were cultivated under shaking conditions at 30 °C or 37 °C for 48 h. The M9 minimal medium used in this study consisted of the following chemicals (per liter): 6.78 g Na_2_HPO_4_, 3 g KH_2_PO_4_, 0.5 g NaCl, 1 g NH_4_Cl, 0.493 g MgSO_4_·7H_2_O, 11 mg CaCl_2_, 0.1 g thiamine, 2.78 mg FeSO_4_·7H_2_O, 2.41 mg MnSO_4_·5H_2_O, 1.3 mg CoCl_2_, 1.36 mg ZnCl_2_, 2.42 mg Na_2_MoO_4_·2H_2_O, 1.7 mg CuCl_2_·2H_2_O, and 0.62 mg H_3_BO_3_. For the time-course analysis of P(3HB) production, the transformants were inoculated into 150 mL of M9 minimal medium in 500 mL baffled Erlenmeyer flasks. Cell turbidity was measured spectrophotometrically at an absorbance of 660 nm (UV-1200; Shimadzu Corporation, Kyoto, Japan).

### Analysis of cellular poly(3-hydroxybutyrate)

P(3HB) accumulation in the transformants was analyzed according to the method described by Ishizaki and Tanaka [41]. After cultivation, the cells were collected by centrifugation at 5,800×*g* (4 °C) and were washed with 10 mL of 0.85% (w/v) NaCl solution. The recovered cells were suspended in 10 mL of acetone and allowed to stand overnight. After removing acetone by centrifugation, the dried *E. coli* cells were prepared by reduced-pressure drying. The dried cells were resuspended in 2 mL of chloroform and subjected to methanolysis at 100 °C for 3.5 h after the addition of 2 ml of 3% (v/v) H_2_SO_4_-methanol. After cooling, 1 mL of distilled water was added to the suspension, which was stirred for 10 min. The solution was divided into two layers by centrifugation and the 3HB methyl ester in the lower chloroform layer was analyzed using gas chromatography with a flame ionization detector (GC-FID), GC-2014 (Shimadzu Co, Kyoto, Japan). A glass packed column (3 m × 4 mm diameter) filled with 2% Reoplex 400 on Chromosorb GAW 60‒80 mesh (Shimadzu GLC Ltd, Tokyo, Japan) was used. The packed column was purged with nitrogen gas at 40 mL/min, hydrogen at 60 kPa, and air at 50 kPa. The injector and detector temperatures were maintained at 180 °C. The elution conditions were as follows: 90 °C for 1 min, 90 °C–140 °C at 8 °C increase/min, and 140 °C for 5 min. The cellular P(3HB) content was calculated as mg of P(3HB) per mg of dry cell weight, and expressed in % (w/w).

### Analysis of metabolites by capillary electrophoresis-mass spectrometry

Following overnight preculture in LB medium, the transformants were grown in minimal medium supplemented with 2 mM pantothenate or β-alanine at 37 °C with shaking. After a 32-h cultivation, 5 mL of culture broth were centrifuged at 5,800 × *g* (4 °C) for 5 min and the collected cells were washed with 5 mL of 0.85% (w/v) NaCl. The cells were collected by centrifugation and resuspended in 5 mL of 0.8% (w/v) NaCl containing 40% (v/v) ethanol. After centrifugation, the cell pellet was resuspended in 0.75 mL ethanol (HPLC grade) containing 53.33 μM L-methionine sulfone, 53.33 μM PIPES, and 6.67 μM D-camphor-10-sulfonic acid and incubated at 70 °C for 15 min, followed by standing on ice for 2 min. To extract the metabolites, 0.75 mL of Milli Q water were added to the suspension followed by 1 mL of chloroform (HPLC grade). After mixing well and centrifugating at 5800×*g* (4 °C) for 5 min, the water layer (0.5 mL) was transferred to an ultrafilter membrane unit (Amicon Ultra-0.5 Centrifugal Filter Unit) and centrifuged at 15100×*g* (4 °C) for 90 min. The resulting filtrate (300 μL) was evaporated, and the dried samples were stored at − 80 °C until use. After being dissolved in water, the extracted metabolites were subjected to CE-MS, as described previously [42].

### Measurement of residual glucose in the culture medium

The residual glucose concentration in the culture media was measured by high-pressure liquid chromatography equipped with a refractive index detector (RI-2031, Jasco Corporation, Japan). A Shodex Super sugar SH1011 column (8.0 mm diameter × 300 mm, Showa Denko K.K., Japan) was equilibrated in advance with 5 mM H_2_SO_4_ and was maintained at 25 °C. Elution was done at a flow rate of 0.6 mL/min.

### Statistical analysis

Tukey's honestly significant difference (HSD) test was performed for the analyses of statistical differences in P(3HB) accumulation and metabolic intermediate concentrations. The effect of the introduction of *coaA* in the presence of various concentrations of CoA precursors on P(3HB) production was compared using an unpaired Student's *t*-test. The statistical analyses were performed using R version 4.2.1 (R Development Core Team) [43].

## Supplementary Information

**Additional file 1: Figure S1.** Confirmation of plasmids carried by the prepared transformants. **Figure S2.** Growth curves of the transformants carrying the *pha* genes from *M. aeruginosa*. **Table S1.** Plasmid retention rates of the *E. coli* transformants during P(3HB) production. **Table S2.** Oligonucleotide primers used in this study. **Figure S3.** Cloning of the *pha* genes from *Microcystis aeruginosa* NIES-843 (A) and sequencing of the genes cloned in pQE-60, designated pQE-Ma-phaABEC (B).

## Data Availability

All data generated or analyzed during this study are included in this published article.

## Abbreviations

3HB: 3-Hydroxybutyric acid
Acc: Acetyl-CoA carboxylase
AckA: Acetate kinase
Ap: Ampicillin
GC-FID: Gas chromatography with a flame ionization detector
CE-MS: Capillary electrophoresis-mass spectrometry
Cm: Chloramphenicol
CoaA: Pantothenate kinase
HPLC: High performance liquid chromatography
IPTG: Isopropyl β-D-thiogalactopyranoside
P(3HB): Poly(3-hydroxybutyrate)
PanC: Pantothenate synthase
PanD: L-aspartate-α-decarboxylase
PHA: Polyhydroxyalkanoate
PhaA: β-Ketothiolase
PhaB: Acetoacetyl-CoA reductase
PhaEC: Polyhydroxyalkanoate synthase
PoxB: Pyruvate oxidase
Pta: Phosphate acetyltransferase
