# Supplementary material for: Enhanced supply of acetyl-CoA by exogenous pantothenate kinase promotes synthesis of poly(3-hydroxybutyrate)
Source: Microb Cell Fact. 2023 Apr 20;22:75. doi: 10.1186/s12934-023-02083-5 (PMC10116679; doi:10.1186/s12934-023-02083-5)
Supplement: Supplementary file 1 — Additional file 1: Figure S1. Confirmation of plasmids carried by the prepared transformants. Figure S2. Growth curves of the transformants carrying the pha genes from M. aeruginosa. Table S1. Plasmid retention rates of the E. coli transformants during P(3HB) production. Table S2. Oligonucleotide primers used in this study. Figure S3. Cloning of the pha genes from Microcystis aeruginosa NIES-843 (A) and sequencing of the genes cloned in pQE-60, designated pQE-Ma-phaABEC (B). [file 12934_2023_2083_MOESM1_ESM.docx]

**Figure S1.** Confirmation of plasmids carried by the prepared transformants. PCR was conducted using the primer sets of Pp-coaA-cpF1 and Pp-coaA-cpR1 for pSTV-Pp-coaA (panel A, odd-numbered lanes in panel C) and Ma-phaE-cpF1 and Ma-phaE-cpR1 for pQE-Ma-phaABEC (panel B, even-numbered lanes in panel C), respectively. The plasmids purified from each transformant were used as the template DNA. The amplified DNA fragments were analyzed with 2% agarose gel electrophoresis (panel C). Strain no.1 held pSTV28 and pQE-60; no.2, pSTV-Pp-coaA and pQE-60; no.3, pSTV28 and pQE-Ma-phaABEC; no.4, pSTV-Pp-coaA and pQE-Ma-phaABEC. P*lac*, *lac* promoter; PT5, T5 promoter; *ori*, origin of replication.

**Figure S2.** Growth curves of the transformants carrying the *pha* genes from *M. aeruginosa*. *E. coli* JM109/pSTV28 + pQE-Ma-phaABEC (closed symbols) and JM109/pSTV-Pp-coaA + pQE-Ma-phaABEC (open symbols) were cultivated in M9 minimal medium containing 2% (w/v) glucose, 5 mM pantothenate, 100 μg/mL Ap, and 25 μg/mL Cm at 37 °C for 48 h. The growth curves in the absence or presence of 0.1 mM IPTG were indicated in circles and triangles, respectively.

**Table S1.** Plasmid retention rates of the *E. coli* transformants during P(3HB) production

| **Culture media** | **Plasmid retention rate (%)** | | | |
| --- | --- | --- | --- | --- |
|  | ***E. coli* JM109/pSTV28 + pQE-Ma-phaABEC** | | ***E. coli* JM109/pSTV-Pp-coaA + pQE-Ma-phaABEC** | |
|  | **IPTG (-)** | **IPTG (+)** | **IPTG (-)** | **IPTG (+)** |
| LB-Ap | 100 | 72 | 100 | 41 |
| LB-Cm | 100 | 100 | 99 | 98 |
| LB-Ap + Cm | 100 | 72 | 99 | 41 |

*E. coli* JM109/pSTV28 + pQE-Ma-pfaABEC and JM109/pSTV-Pp-coaA + pQE-Ma-phaABEC were aerobically cultivated in M9 minimal medium supplemented with 2% (w/v) glucose, 5 mM pantothenate, 100 μg Ap, and 25 μg/mL Cm at 37 °C for 48 h. An aliquot of culture medium diluted with LB medium was spread over an LB agar plate. After incubation at 37 °C for 24 h, 100 single colonies were picked off the LB plates containing 100 μg/mL Ap and/or 25 μg/mL Cm and the inoculated plates were maintained at 37 °C for 24 h. The plasmid retention rates were calculated by counting the transformants appearing on the plates.

**Table S2.** Oligonucleotide primers used in this study

| **Primer** | **Nucleotide sequence (5′ to 3′)** |
| --- | --- |
| Ma-phaA-gF | GAGAAATTAACCATGATCTTTTCGGACTCGATATTCTTAAGC |
| Ma-phaC-gR | CTGGATCCTCCCATGTTAAGCTCTTGCTTTCAACCAATCGCTAAT |
| Ma-phaA-485F | GTGCGCCGGAACAATTAAC |
| Ma-phaA-1002F | GATCGAAGATTTCGATTTAGTGG |
| Ma-phaB-1542F | CCCTAAATTAACGCCGCTAG |
| Ma-phaBE-2038F | GGTGTAGGGTGTAGGGTGGGTTAGTAAAC |
| Ma-phaE-2653F | GAGAATTTAACCGCAAATTACTC |
| Ma-phaEC-3182F | GTGTTAGGTGAAAACTTCGGAAC |
| Ma-phaC-3741F | CCTTTAGTTTATGCTATAGTTCCCTC |
| Ma-phaA-429R | GGACATGGATTCAATACCACC |
| Ma-phaE-cpF1 | ATGGATAAACCGACACAAGCTTG |
| Ma-phaE-cpR1 | CTAATTGTTACTTTCTACTGCTTTTTTTTC |
| Pp-coaA-cpF1 | ATGATTCTTGAGCTCGATTGCG |
| Pp-coaA-cpR1 | TCAATCCAATGGGCAGGCCAT |
| pQE-PR | CCCGAAAAGTGCCACCTG |

**Figure S3.** Cloning of the *pha* genes from *Microcystis aeruginosa* NIES-843 (A) and sequencing of the genes cloned in pQE-60, designated pQE-Ma-phaABEC (B).
